# Systemic Bacillus Calmette-Guerin Infection One Year After Intravesical Immunotherapy Mimicking Sarcoidosis

**DOI:** 10.7759/cureus.31697

**Published:** 2022-11-20

**Authors:** Yashswee Kc, Mohit Gupta, Gail E Reid, Ayesha Arif, Elaine Adams

**Affiliations:** 1 Rheumatology, Loyola University Medical Center, Maywood, USA; 2 Internal Medicine, Loyola University Medical Center, Maywood, USA; 3 Infectious Disease, Edward Hines, Jr. VA (Veterans Affairs) Hospital, Hines, USA; 4 Rheumatology, University of Vermont, Burlington, USA; 5 Rheumatology, Edward Hines, Jr. VA (Veterans Affairs) Hospital, Hines, USA

**Keywords:** non-caseating granuloma, severe hypercalcemia, m. bovis, intravesical immunotherapy, sarcoidosis “like, bacillus calmette-guerin infection

## Abstract

A patient presented with pancytopenia and hypercalcemia after intravesical immunotherapy with Bacillus Calmette-Guerin (BCG) for bladder cancer. Bone marrow biopsy performed six months later revealed noncaseating granulomas with negative stains for AFB. He was diagnosed with sarcoidosis and treated with prednisone. Hypercalcemia resolved, but mild pancytopenia persisted. One year later, he developed sepsis. Blood cultures six weeks later grew Mycobacterium tuberculosis complex, ultimately identified as Mycobacterium bovis. Despite triple antibiotic therapy, the patient progressively declined and expired.

## Introduction

Granulomas are focal aggregates of macrophages, epitheliod cells, multinucleated giant cells, and lymphocytes that form in response to persistent inflammatory or infectious stimuli [1]. Common organ involvement includes the liver, lung, and bone marrow, but granulomas can be present almost anywhere. Multiple etiologies have been described for bone marrow granulomas, including malignancy and infectious diseases (brucellosis, typhoid fever, Q fever, and mycobacterium). Sarcoidosis and autoimmune diseases are less common causes [2].

One rare cause of granuloma is disseminated Bacillus Calmette-Guerin (BCG) infection. BCG is a vaccine developed from the bacterium Mycobacterium bovis (M. bovis). This organism was distinguished from Mycobacterium tuberculosis (MTB) by Robert Koch in early attempts to develop a vaccine against tuberculosis [3]. Calmette and Guerin later developed an attenuated strain of M. bovis (BCG) that stimulated the immune response upon administration [4,5]. BCG treatment is used as an adjuvant to cancer immunotherapy, including noninvasive bladder cancer [5-7]. Administration of BCG into the bladder is widely utilized after bladder cancer resection with a reduction in cancer recurrence of up to 70% [4]. Despite the low-level virulence of BCG, clinical M. bovis infection after intravesical administration has been reported [6,8,9].

## Case presentation

A 69-year-old male presented to the hospital with recurrent episodes of hypercalcemia. He had a past medical history of head and neck squamous cell carcinoma status post resection and adjuvant cisplatin chemotherapy completed one year prior to admission and bladder cancer status post transurethral resection of bladder tumor followed by BCG intravesical immunotherapy completed six months prior to admission. The final BCG administration was complicated by extensive gross hematuria and a prolonged flu-like illness that lasted for weeks. Upon admission, he complained of nonspecific fatigue and weakness but no weight loss, nausea, vomiting, polyuria, abdominal pain, myalgia, or arthralgia. His temperature was 98.4, respiratory rate 18, blood pressure 163/80, pulse 66, and pulse oximetry 96% on room air. The physical exam was significant for a chronically ill-looking patient with mild pallor. He had no lymphadenopathy. His cardiovascular and respiratory system examinations were unremarkable. His abdominal examination revealed a soft, nontender abdomen with no hepatosplenomegaly, soft in consistency.

Laboratory evaluation revealed pancytopenia with WBC 3.67 K/uL, hemoglobin (Hgb) 9.6 M/uL, and platelet 107 K/uL. His creatinine (Cr) was elevated at 1.94 mg/dL. Urinalysis didn't reveal proteinuria or hematuria. Calcium was elevated at 11.4 mg/dL. Parathyroid hormone (PTH) was appropriately low at 8.5 pg/dL, and parathyroid hormone-related protein (PTH-rP) was also low at 13 pg/mL. He had normal 25-OH Vit D with mildly elevated 1-25 vitamin D level at 77 pg/mL (normal 18-72). He had a normal angiotensin-converting enzyme (ACE) level of 55 U/L (Table 1). Serum protein electrophoresis (SPEP), urine protein electrophoresis (UPEP), and serum-free light chains were normal (Table 1).

**Table 1:** Laboratory information PTH: parathyroid hormone; PTH-rP: parathyroid hormone-related protein; ACE: angiotensin-converting enzyme

Laboratory Test	Normal Range	Result
Hemoglobin, M/uL	13-17	9.6
White blood cells, K/uL	4-11	3.67
Platelet count, K/uL	130-400	107
Calcium mg/dL	13.2-16.6	11.4
Vitamin D ng/mL	30-100	50
1-25 Vitamin D, pg/mL	18-72	77
PTH, pg/dL	18.4-80.1	8.5
PTH-rP pg/mL	14-27	13
ACE U/L	9-67	55
Creatinine mg/dL	0.67-1.17	1.94

**Figure 1:**
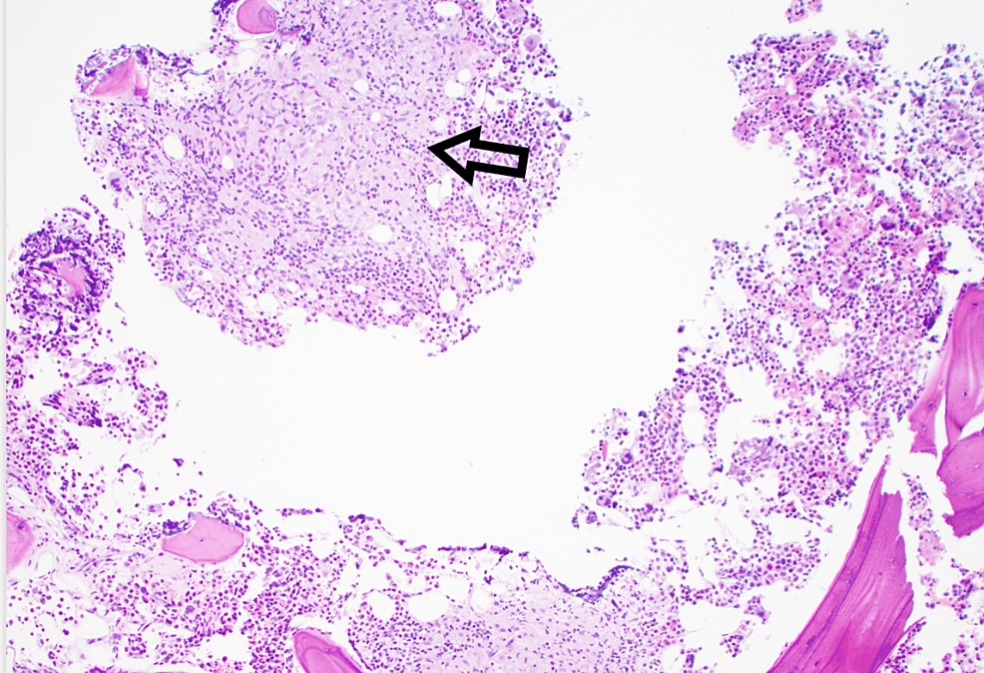
H&E-stained bone marrow biopsy with non-caseating epithelioid granulomas H&E: hematoxylin and eosin

CT scan of the neck, chest, abdomen, and pelvis revealed mild hilar adenopathy but no recurrence of cancer. There was a small, calcified granuloma in the liver, which was not present on prior abdominal imaging. A skeletal survey revealed no destructive bone lesions. A bone marrow biopsy showed noncaseating granulomas without evidence of dysplasia or plasmacytoid process (Figure 1). AFB stains were negative. A kidney biopsy revealed nephrosclerosis but no granulomas. Based on hilar lymphadenopathy, mildly elevated 1-25 vitamin D level and granulomas on bone marrow biopsy, the patient was given a diagnosis of sarcoidosis. He was started on prednisone 60 mg for four weeks followed by prednisone 40 mg daily. Calcium levels returned to normal, and the prednisone was slowly tapered eventually to 10 mg daily. Given the patient's persistent pancytopenia, a decision was made to add a steroid-sparing agent. Mycophenolate was chosen, as this medication would be less likely to carry the additional potential for bone marrow suppression that can be seen with azathioprine and methotrexate. 

One year later, the patient returned to the hospital with sepsis-like syndrome along with hypotension, fever, and persistent pancytopenia. Repeat bone marrow biopsy again showed non-caseating granulomas with negative AFB stain. Blood, urine, bone marrow, and sputum cultures were negative. Six weeks after his initial presentation, blood cultures for AFB became positive for Mycobacterium complex, subsequently confirmed to be M. bovis. Prednisone and mycophenolate given for sarcoid were discontinued, and he was started on isoniazid, ethambutol, rifabutin, and vitamin B6. Despite antibiotic therapy, the patient continued to deteriorate and expired one month later. The final diagnosis was systemic BCG infection 18 months after intravesical administration of BCG based on positive blood cultures for M. bovis and demonstration of granulomas in bone marrow and liver.

## Discussion

BCG is a live attenuated strain of M. bovis. Intravesical administration is usually safe, however, local and systemic infections can occur [6]. In the series of Lamm et al., disseminated BCG infection developed in less than 1% of patients, granulomatous hepatitis in 0.7%, and cytopenia in 0.1% [10]. Traumatic catheterization, advanced age, and immunosuppression are risk factors for the hematogenous and lymphatic spread of the attenuated BCG strain [11]. Systemic complications from BCG administration include sepsis, mycotic aneurysms, pneumonitis, hepatitis, and osteoarticular infections. Rare cases of bone marrow, central nervous system, and ocular involvement have also been reported [6]. A disseminated infection has a high mortality rate of 70% [8].

M. bovis-related symptoms may appear shortly after BCG administration or years later, making the diagnosis difficult. The finding of granulomas on a biopsy of involved organs is nonspecific, and AFB stains can be negative despite the presence of mycobacterium [12]. The slow growth of Mycobacteria and fastidious culture requirements further confound this challenging diagnosis. In our patient, multiple cultures were taken before mycobacterium was identified in the blood culture, six weeks after collection.

The typical presentation of disseminated BCG infection includes fever, malaise, and pancytopenia, which occur in many other conditions. The finding of granulomas can easily point to sarcoidosis as a more likely diagnosis, as in our patient. Hypercalcemia has not previously been reported as a complication of M. bovis infection. We postulate that hypercalcemia was mediated by an increase in calcitriol as documented in other granulomatous diseases [13].

## Conclusions

In summary, although BCG is generally safe, disseminated M. bovis disease is a rare and serious adverse reaction that can occur following BCG vaccination. Symptoms of the infection can present one or more years after BCG administration. A high index of suspicion is required. The confirmation also becomes difficult due to M. bovis only growing in selective culture media under special laboratory conditions and at a very slow rate. A misdiagnosis with another granulomatous condition, such as Sarcoidosis, can occur given the similar clinical presentation with the presence of granulomas. A range of clinical presentations can occur. We add bone marrow involvement and hypercalcemia to the spectrum.

## References

[1] Wang Y, Tang XY, Yuan J (2018). Bone marrow granulomas in a high tuberculosis prevalence setting: a clinicopathological study of 110 cases. Medicine (Baltimore).

[2] Eid A, Carion W, Nystrom JS (1996). Differential diagnoses of bone marrow granuloma. West J Med.

[3] Med-Rath G, Koch R (2007). The combating of tuberculosis in the light of the experience that has been gained in the successful combating of other infectious diseases. J Laryngol Otol.

[4] Guallar-Garrido S, Julián E (2020). Bacillus Calmette-Guérin (BCG) therapy for bladder cancer: an update. Immunotargets Ther.

[5] Luca S, Mihaescu T (2013). History of BCG vaccine. Maedica (Bucur).

[6] Liu Y, Lu J, Huang Y, Ma L (2019). Clinical spectrum of complications induced by intravesical immunotherapy of Bacillus Calmette-Guérin for bladder cancer. J Oncol.

[7] Nishida S, Tsuboi A, Tanemura A (2019). Immune adjuvant therapy using Bacillus Calmette-Guérin cell wall skeleton (BCG-CWS) in advanced malignancies: A phase 1 study of safety and immunogenicity assessments. Medicine (Baltimore).

[8] Robinson AR, Radhakrishnan R, Horvath R, Pandey S (2017). A case of disseminated Mycobacterium bovis 2 years post-intravesicular Bacillus Calmette-Guérin therapy for superficial urinary bladder cancer. Intern Med J.

[9] Parent ME, Richer M, Liang P (2018). The first case of bacillus Calmette-Guérin-induced small-vessel central nervous system vasculitis. Clin Rheumatol.

[10] Lamm DL, van der Meijden PM, Morales A (1992). Incidence and treatment of complications of bacillus Calmette-Guerin intravesical therapy in superficial bladder cancer. J Urol.

[11] Brake M, Loertzer H, Horsch R (2000). Long-term results of intravesical Bacillus Calmette-Guérin therapy for stage T1 superficial bladder cancer. Urology.

[12] Safdar N, Abad CL, Kaul DR, Jarrard D, Saint S (2008). Clinical problem-solving. An unintended consequence--a 79-year-old man with a 5-month history of fatigue and 20-lb (9-kg) weight loss presented to his local physician. N Engl J Med.

[13] Adams JS, Gacad MA, Anders A, Endres DB, Sharma OP (1986). Biochemical indicators of disordered vitamin D and calcium homeostasis in sarcoidosis. Sarcoidosis.

